# BCAAs acutely drive glucose dysregulation and insulin resistance: role of AgRP neurons

**DOI:** 10.1038/s41387-024-00298-y

**Published:** 2024-06-06

**Authors:** Harsh Shah, Ritchel B. Gannaban, Zobayda Farzana Haque, Fereshteh Dehghani, Alyssa Kramer, Frances Bowers, Matthew Ta, Thy Huynh, Marjan Ramezan, Ashley Maniates, Andrew C. Shin

**Affiliations:** Neurobiology of Nutrition Laboratory, Department of Nutritional Sciences, College of Human Sciences, Texas Tech University, Lubbock, TX USA

**Keywords:** Obesity, Obesity

## Abstract

**Background:**

High-protein diets are often enriched with branched-chain amino acids (BCAAs) known to enhance protein synthesis and provide numerous physiological benefits, but recent studies reveal their association with obesity and diabetes. In support of this, protein or BCAA supplementation is shown to disrupt glucose metabolism while restriction improves it. However, it is not clear if these are primary, direct effects of BCAAs or secondary to other physiological changes during chronic manipulation of dietary BCAAs.

**Methods:**

Three-month-old C57Bl/6 mice were acutely treated with either vehicle/BCAAs or BT2, a BCAA-lowering compound, and detailed in vivo metabolic phenotyping, including frequent sampling and pancreatic clamps, were conducted.

**Results:**

Using a catheter-guided frequent sampling method in mice, here we show that a single infusion of BCAAs was sufficient to acutely elevate blood glucose and plasma insulin. While pre-treatment with BCAAs did not affect glucose tolerance, a constant infusion of BCAAs during hyperinsulinemic–euglycemic clamps impaired whole-body insulin sensitivity. Similarly, a single injection of BT2 was sufficient to prevent BCAA rise during fasting and markedly improve glucose tolerance in high-fat-fed mice, suggesting that abnormal glycemic control in obesity may be causally linked to high circulating BCAAs. We further show that chemogenetic over-activation of AgRP neurons in the hypothalamus, as present in obesity, significantly impairs glucose tolerance that is completely normalized by acute BCAA reduction. Interestingly, most of these effects were demonstrated only in male, but not in female mice.

**Conclusion:**

These findings suggest that BCAAs per se can acutely impair glucose homeostasis and insulin sensitivity, thus offering an explanation for how they may disrupt glucose metabolism in the long-term as observed in obesity and diabetes. Our findings also reveal that AgRP neuronal regulation of blood glucose is mediated through BCAAs, further elucidating a novel mechanism by which brain controls glucose homeostasis.

## Introduction

The rising prevalence of obesity and diabetes across the globe is leading individuals to seek various dietary, pharmacological, and/or surgical approaches to maintain or restore their health. In particular, more people today choose to consume diets high in protein content, including that of branched-chain amino acids (BCAAs) [1]. BCAAs (i.e., leucine, isoleucine, and valine) are essential amino acids we need to obtain from diet sources such as meat, fish, dairy, and legumes. The proclivity toward diets rich in BCAAs is not surprising due to their important role in hormone regulation [2–4], fat metabolism [5, 6], immune-boosting effects [7, 8] and physical fitness [9–11]. BCAA supplementation is also widely used in clinical settings for preventing muscle wasting in patients with liver cirrhosis, kidney failure, cancers, or sepsis [12–17].

Paradoxically, however, circulating BCAAs are found to be consistently elevated in obesity, insulin resistance, and Type 2 diabetes (T2D) [18–26], and they are the earliest predictive marker for future risk of diabetes [27]. Moreover, BCAA supplementation has been shown to induce glucose dysregulation and insulin resistance [21, 28, 29], whereas reducing plasma BCAA levels by dietary [28, 30, 31], pharmacological [32, 33], or surgical [34–36] interventions can lead to significant improvement in insulin sensitivity and glycemic control. Therefore, it is critical to elucidate the mechanism by which BCAAs disrupt glucose metabolism that can help establish a safe limit of BCAA intake for individuals.

The metabolic perturbation via BCAA supplementation as well as the benefits of BCAA restriction/deprivation demonstrated thus far correspond to long-term effects, making it difficult to determine if these outcomes are primary effects of BCAAs or secondary due to concurrent changes in relevant parameters, including energy balance, inflammation, and liver functions [21, 23, 37]. Determining the acute effects of BCAAs on glucose regulation seems necessary to establish a direct causal relationship. Ikehara and colleagues [38] has shown that a single gavage of isoleucine in normal mice improves glucose tolerance. However, because elevating the plasma level of one BCAA can alter the levels of the other two [39, 40], and most BCAA-rich dietary sources often contain a ratio of all BCAAs and not an isolated one, investigating the role of whole BCAAs on glucose homeostasis is deemed more physiological and clinically relevant.

To this end, this study examined the acute effects of BCAA exposure or breakdown on glycemic control and insulin sensitivity in both male and female mice. A number of in vivo physiological techniques such as catheter-guided frequent sampling and glucose tolerance test were conducted to assess glucose homeostasis, and insulin tolerance test and hyperinsulinemic–euglycemic clamp were employed to determine insulin sensitivity following acute exposure to BCAAs or its catabolism. To understand the effect of BCAA manipulation at different nutritional status, the role of acute BCAAs was assessed in mice on either a regular chow or high-fat diet. We recently demonstrated that agouti-related protein (AgRP) neurons in the hypothalamus can disrupt glucose homeostasis and elevate plasma BCAAs [41]. To determine if high BCAAs are necessary in mediating impaired glycemia, we chemogenetically stimulated AgRP neurons in mice treated with a BCAA-lowering compound and assessed glucose metabolism.

## Materials and methods

### Animals

3-month-old male and female C57BL/6J mice (Stock No. 000664, Jackson Laboratory) were used for all the experiments that assessed the acute effects of BCAAs on glucose homeostasis and insulin sensitivity. These mice were group-housed and had ad libitum access to a regular chow diet (Lab diet, 5001) and water. Another cohort of C57BL/6J mice were placed on a 60% HF diet (58Y1, Lab diet) for 8 weeks before treatment with vehicle or 3,6-dichlorobenzo[b]thiophene-2-carboxylic acid (BT2). Their fat and lean mass were analyzed by EchoMRI prior to the experiment. For the chemogenetic DREADD experiment, 3-month-old Agrp^tm1(cre)Lowl^/J (Stock no. 012899, Jackson Laboratory) and C57BL/6J WT mice were used. AgRP-IRES-Cre mice were bred in our animal facility at Texas Tech University. Mice were housed on a 12 h:12 h cycle (7:00 am–7:00 pm). Experimental procedures were conducted in either the animal facility procedure room or in the investigator’s laboratory. For each experiment, age, sex, and body weight were matched between control and treatment groups. All studies were conducted in accordance with the National Institutes of Health’s Guide for the Care and Use of Laboratory Animals, and the protocol was approved by the Institutional Animal Care and Use Committee (IACUC) at Texas Tech University.

### Dose of BCAAs and BT2

With the average protein intake in the US being ~1.7 g/kg BW/day, it contains about 132, 75, and 90 mg/kg BW of leucine, isoleucine, and valine (2.0:1.14:1.36), respectively [42]. The proportion of BCAAs in the chow diet used here (Lab diet #5001) is 2.0:1.13:1.22 (Leu:Ile:Val). Hence, we rounded up this ratio for a simple calculation to accomplish 2:1:1. We used a BCAA dose of 2.25 mmole/kg BW for all our experiments. This is based on the fact that the mean single meal size of a mouse weighing 30 g is ~0.216 g which corresponds to approximately 2.25 mmoles of BCAAs/kg [43]. When infused directly in the circulation, we used 150 mM of BCAAs prepared in saline (~450 mOsm/l) to minimize any osmotic shock. For intraperitoneal (i.p.) treatment, we injected 10 µl/g BW of 225 mM BCAAs made in saline. 3,6-dichlorobenzo[b]thiophene-2-carboxylic acid (BT2) was dissolved in DMSO and then added to the mixture of Cremophor EL and 0.1 M sodium bicarbonate (pH 9.0), making the final concentration of BT2 to be 4 mg/mL in 5% DMSO, 10% Cremophor EL and 85% of 0.1 M sodium bicarbonate [32]. The vehicle contained the same proportion of DMSO, Cremophor EL, and sodium bicarbonate.

### Jugular catheter implantation

Mice were anesthetized with isoflurane via the isoflurane/oxygen combo + vaporizer apparatus. They were placed on a heating pad to maintain body temperature and were injected with extended-release buprenorphine subcutaneously (1 mg/kg sc) before the surgery started. After exposing and isolating the jugular vein, an indwelling silastic catheter (1.1 cm length; 0.30 mm inner diameter) was inserted into the jugular vein. The catheter was filled with 20 U/ml heparin solution and braided silk sutures were used to secure the catheter in the vein and to close the skin. Then, the indwelling catheter was tunneled through the back of the neck after which the incision in the back was sutured. At the end of the surgery, the mice were allowed to recover on the heating pad under observation, and sterile saline was injected subcutaneously for hydration. Body weight and catheter patency were checked every day. Animals with intact catheters after recovery were used for experiments.

### Stereotaxic injection in the brain

Under the isoflurane-induced anesthesia, mice were secured on a digital mouse stereotaxic instrument (#51730D, Stoelting Co.) with an ear bar on each side and securing their front teeth on the teeth holder to hold the cranium in place. C57BL/6J WT or AgRP-IRES-Cre mice received extended-release buprenorphine subcutaneously (1 mg/kg) prior to the surgery. A micro-syringe (#7647-01, Hamilton Company) was filled with 1 µl of AAV8 containing pAAV-hSyn-DIO-hM3D(Gq)-mCherry (#44361-AAV8, Addgene), a stimulatory Designer-Receptors-Exclusively-Activated-by-Designer-Drugs (DREADD). In total, 400 nl of AAV were delivered to the coordinates of the mediobasal hypothalamus (MBH) comprising −1.6 mm distance from anterior to posterior (AP), 5.8 mm Dorso-ventral (DV), and 0.25 mm unilaterally to the left at a rate of 100 nl/min using 33-gauge needle (#7762-06, Hamilton Company). The needle was left inside for 5 min to ensure enough diffusion. The skull was disinfected with 70% alcohol using a cotton tip, and the skin was glued using a 3 M Vetbond Tissue Adhesive (#NC0735004, Fisher Scientific). The mice were removed from the stereotaxic instrument and recovered on a heating pad under observation. In all, 1 ml sterile saline was injected subcutaneously for hydration. After surgery, all animals were kept single-housed for experiments.

### Functional validation of hypothalamic DREADD expression

Voracious food intake was used as a surrogate marker of AgRP neuronal activation induced by functional DREADD. After 2–3 weeks of recovery from stereotaxic surgery, male mice at the fed state received clozapine-N-oxide (CNO; 1 mg/kg i.p.) in the morning between 8 am and 9 am to stimulate AgRP neurons. Food intake of a regular chow diet was measured at 0.5, 1, 2, and 3 h post-CNO administration. Mice were transferred back to the home cages afterward until the experiment.

### Hyperinsulinemic–euglycemic clamps

Male and female C57BL/6J mice were subjected to jugular catheter implantation before hyperglycemic euglycemic clamps. On the clamp day, after 2 h of fasting, the catheter was hooked up with a connector that is attached to saline/BCAAs, glucose, and insulin infusion pumps. Baseline blood was collected, and glucose was measured at *t* = −60 min At *t* = 0 min, either vehicle (*n* = 7) or BCAAs (150 mM; 15 µl/g of BW; *n* = 7) was infused at 2 µmol/min for 10 min and switched to 0.2 µmol/min to maintain a constant, high plasma BCAAs during the clamps. Concurrently, a bolus of insulin (1U/ml in saline mixed with donor plasma; Humulin R-100) was infused at a rate of 70 mU/Kg/min for 1 min followed by 3 mU/kg/min for the remainder of the clamp period. Blood glucose was monitored every 10 min by a hand-held glucometer (Alpha Trak 2, Zoetis), and physiological blood glucose (120–150 mg/dl) was maintained by adjusting a variable infusion rate of 25% glucose solution. A steady state was ascertained when blood glucose was stable for at least 30 min at a fixed glucose infusion rate (GIR) and was achieved between *t* = 90–120 min. Blood samples (20–30 µl) were collected during the clamp period at *t* = 0, 30, 60, 90, and 120 min. Plasma was separated from the blood and stored at −80 °C until plasma BCAA and insulin analysis.

### Frequent glucose monitoring

After 4 h fasting, a jugular catheter implanted in male and female mice was infused with either saline (*n* = 8) or BCAAs (150 mM; 2.25 mmol/kg BW; *n* = 10) over 4 min. The mice were all single-housed during the experiment. Blood glucose was measured by a hand-held glucometer, and blood was collected at *t* = 0, 10, 15, 20, 25, 30, 35 min through the catheter. Plasma was separated from the blood for analysis of plasma BCAAs and insulin.

### Glucose tolerance test (GTT)

For normal chow diet experiment, male and female C57BL/6 J mice under regular chow were fasted for 5 h. They were then injected with either saline (males: *n* = 6; females: *n* = 7) or BCAAs (males: *n* = 6; females: *n* = 8) at *t* = −15 min before undergoing GTT with an injection of glucose (15% glucose; 1.5 g/kg BW i.p.) at *t* = 0 min. Blood was collected in Microvette CB300 K2 EDTA tubes, and blood glucose was measured using a hand-held glucometer at *t* = 0, 15, 30, 60, 90 min for female mice and an additional *t* = 120 min for male mice. For male and female mice placed on 8 weeks of HF diet, either saline (males: *n* = 5; females: *n* = 5) or BT2 (40 mg/kg i.p.; males: *n* = 5; females: *n* = 5) was given at *t* = −60 min, after which mice were injected with glucose (1.5 g/kg BW i.p.) at *t* = 0 min. Blood was collected in Microvette CB300 K2 EDTA tubes, and blood glucose was measured using a hand-held glucometer at *t* = −60, 0, 15, 30, 60, 90, and 120 min. WT and AgRP Cre mice fasted for 16 h were first injected with CNO (1 mg/kg i.p.) at *t* = −120 min followed by either vehicle or BT2 (*n* = 6–7/group) at *t* = −60 min and another CNO at *t* = 0 min before GTT. At the end of GTT, plasma was separated by centrifugation at 5000× *g* for 5 min. Plasma was stored at −80 °C until analysis.

### Insulin tolerance test (ITT)

Male and female C57BL/6J mice under a regular chow diet were fasted for 4 h before being injected with either saline (males: *n* = 5; females: *n* = 6) or BCAAs (males: *n* = 5; females: *n* = 6) at *t* = −15 min They were injected with insulin (0.75 IU/kg i.p.) at *t* = 0 min. Blood glucose was measured using a hand-held glucometer at *t* = −15, 0, 15, 30, 60, 90, and 120 min, and blood samples were collected in Microvette CB300 K2 EDTA tubes wherever indicated. For HF diet-fed male and female mice, either saline (males: *n* = 5; females: *n* = 5) or BT2 (40 mg/kg i.p.; males: *n* = 5; females: *n* = 5) was given at *t* = −60 min followed by insulin injection (0.75 IU/kg i.p.) at *t* = 0 min. Blood glucose was measured using a hand-held glucometer at *t* = −60, 0, 15, 30, 60, 90, and 120 min. For DREADD experiment, after 6 h fasting, both WT and AgRP Cre mice received CNO (1 mg/kg i.p.) at *t* = −120 min followed by either vehicle or BT2 (40 mg/kg i.p.; *n* = 5–8/group) at *t* = −60 min before a single bolus of insulin (0.75 IU/kg i.p.) at *t* = 0 min. At the end of ITT, plasma was separated by centrifugation at 5000× *g* for 5 min. Plasma was stored at −80 °C until analysis.

### Animal sacrifice and tissue collections

Following insulin treatment in either vehicle or BCAA-injected mice, liver, epididymal white adipose tissue, and quadriceps muscle were harvested and snap-frozen in liquid nitrogen for western blot analysis. Mice after 3 h of fasting received vehicle or BT2 (40 mg/kg i.p.) and were sacrificed 2 h later using isoflurane overdose followed by cervical dislocation. Blood was collected before and after treatment. Tissues, including liver, quadriceps muscle, hypothalamus, cortex, and brainstem, were collected and snap-frozen using liquid nitrogen. At the end of DREADD experiment, AgRP Cre and WT mice received CNO (1 mg/kg i.p.) at a random-fed state between 9 and 10 am and were sacrificed after 1 h using isoflurane overdose followed by cervical dislocation. Blood was collected before and 1 h after CNO injection. Liver and quadriceps muscle were collected and snap-frozen by liquid nitrogen. After harvesting, the brains were washed with ice-cold saline and immediately transferred into a 15-ml tube containing ice-cold 4% paraformaldehyde (PFA) for immunohistochemistry to verify DREADD expression in the hypothalamus.

### Plasma hormone measurement

Insulin, Glucagon, and C-peptide were measured in the plasma by using an insulin ELISA kit (Mercodia, 10-1247-01), glucagon ELISA kit (Mercodia, 10-1281-01), and C-peptide ELISA kit (Crystal Chem, 90050), respectively, as per the manufacturer’s instructions. Plasma corticosterone was measured by corticosterone ELISA kit (Crystal Chem, 80556).

### BCAA enzymatic assay

To measure plasma BCAA levels in mice, a spectrophotometric assay that measures NADH generated from BCAA oxidation was used as previously described [44]. Briefly, seven leucine standards were prepared by adding leucine into ddH_2_O to make concentrations of 200, 400, 600, 800, 1000, and 2000 µM. For 10 ml of reaction buffer, 1 ml of 0.1 M glycine (pH 10.5), 1 ml of 0.1 M KCl (pH 10.5), and 1 ml of 0.1 M KOH (pH 10.5) were added to 7 ml of ddH_2_O. Additionally, for each 10 ml of buffer, 0.1 ml of 0.2 M EDTA (pH 8) was added. The above calculation was scaled up or down depending on the number of samples needed, given that each well of a 96-well plate needs 135 µl of the buffer. Finally, the pH of the solution was kept between 10.5 and 10.7. KOH or HCl were added as appropriate to adjust pH. NAD solution was made by adding 20 mg of NAD to 250 µl of sodium carbonate (pH 10.7). In total, 5 µl of standards or samples was added, followed by 5 µl of freshly prepared NAD to 135 µl of buffer, except the blank. Absorbance 1 was taken at 340 nm afterward. After adding 5 µl of leucine dehydrogenase (200U/ml) to each well that was prepared in 25 mM sodium phosphate buffer with pH 7, the plate was incubated at 37 °C in dark for 30 min, and absorbance 2 was taken at 340 nm. After subtracting the values of absorbance 1 from absorbance 2, the standard curve of BCAA concentrations vs. absorbance was plotted, and BCAA content in the control or treatment group was calculated by using the linear equation “*y*= *m*x + *c*,” where *m* is the slope of the linear graph, and *c* is the value of intercept at the *y* axis.

### Immunohistochemistry

Brains were harvested during euthanasia, and after cleaning with saline, brains were kept in 4% PFA for 24 h at 4 °C. Then, brains were immersed in 30% sucrose solution for 24–48 h and embedded with FSC 22 clear (Leica, 3801480) in a mold and stored at −80 °C until sectioning. Brains were sectioned at 20 µm thickness and mounted on slides. Slides with sections were kept at room temperature for 30 min before proceeding to the next step. Sections were fixed for 10 min in 4% PFA in PBS followed by washing with PBS. Next, sections were kept in 0.25% Triton X (made in PBS) for 10 min followed by washing with PBS. Sections were kept in 1.25 µg/µL DAPI for 1 min and washed with PBS. Coverslips were mounted on sections with Fluoromount-G® (0100-01, Southern Biotech) and sealed with transparent nail polish. DAPI and mCherry were imaged using an Olympus BX-41 microscope.

### Western blot

Liver, quadriceps, white adipose tissue, or brain tissues were homogenized in radioimmune precipitation assay (RIPA) buffer (Cell Signaling, Cat # 9806) with added protease inhibitor (Roche, Cat # 04693132001) in bead tissue lyser (Tissue Lyser LT, Cat#85600) for 5–7 min with an oscillation frequency of 50 Hz. The homogenized mixture was centrifuged in a two-step process to extract the protein (5 min at 2500 × *g* and 15 min at 13,000× *g*). Protein quantification was done by bicinchoninic acid (BCA) protein assay (Thermo Fisher, Cat # 23225). Protein extracts (30 μg) were loaded into gel wells to separate proteins by using sodium dodecyl sulfate-polyacrylamide gel electrophoresis (SDS-PAGE) and then transferred onto a polyvinylidene difluoride (PVDF) membrane using Trans-Blot® Turbo™ Transfer System. The membrane was blocked by 5% non-fat milk in Tris-buffered saline with 1% Tween 20 (TBST) for 1 h, followed by overnight incubation with primary antibodies AKT (Cell Signaling Technology, Cat # 4685), Phospho-AKT (Cell Signaling Technology, Cat # 9271), BCKDHA (Abcam, Cat # ab138460) and Phospho-BCKDHA (Abcam, Cat # 200577). The membrane was washed with TBST and was immunoblotted with an anti-rabbit secondary antibody conjugated with horseradish peroxidase (1:3000 dilution, Cell Signaling, Cat # 7074) for 1 h. After washing the membrane with TBST, Clarity western ECL (Bio-Rad, Cat # 170–5061) was used as a substrate reagent, and chemiluminescence was captured using ChemiDoc Imaging Systems (Bio-Rad Laboratories). Identified protein bands were quantified by ImageJ software.

### Statistical analysis

Sample size per group in each experiment was determined primarily based on the number of animals we used for our pilot studies to reach statistical significance on group mean differences. Any sick animals or AgRP-IRES-Cre mice that did not respond to CNO were not included in the study. Animals were always paired for different treatments in the same setting (i.e., saline vs. BCAAs/BT2) to minimize any variabilities or bias. The experimenters were aware of the group allocations during the conduct of the experiment. Comparisons between two groups were calculated using Welch’s *t* test. For comparisons between three groups or more, Welch’s ANOVA followed by Dunnett’s T3 multiple comparison test was employed. The area of the curve (AOC) for GTT was calculated using the trapezoidal rule. The area under the curve (AUC) was calculated for the individual BCAA injection experiment only because the blood glucose excursion was nearly flat after saline infusion. In addition, two-way Repeated Measures ANOVA followed by post hoc Bonferroni’s correction was used to account for the time and treatment factors on glucose, BCAAs, Insulin, glucagon, C-peptide, and corticosterone. Pre- and post-treatment plasma BCAAs were analyzed by Student’s paired *t* test. All statistical analysis was performed using GraphPad Prism version 9.5.1. Densitometry was analyzed in ImageJ version 1.53k software to determine protein abundance in western blots. All results here are presented as mean ± SEM with statistical significance set at *P* < 0.05.

## Results

### An acute BCAA treatment elevates blood glucose and insulin in male mice

**Fig. 1:**
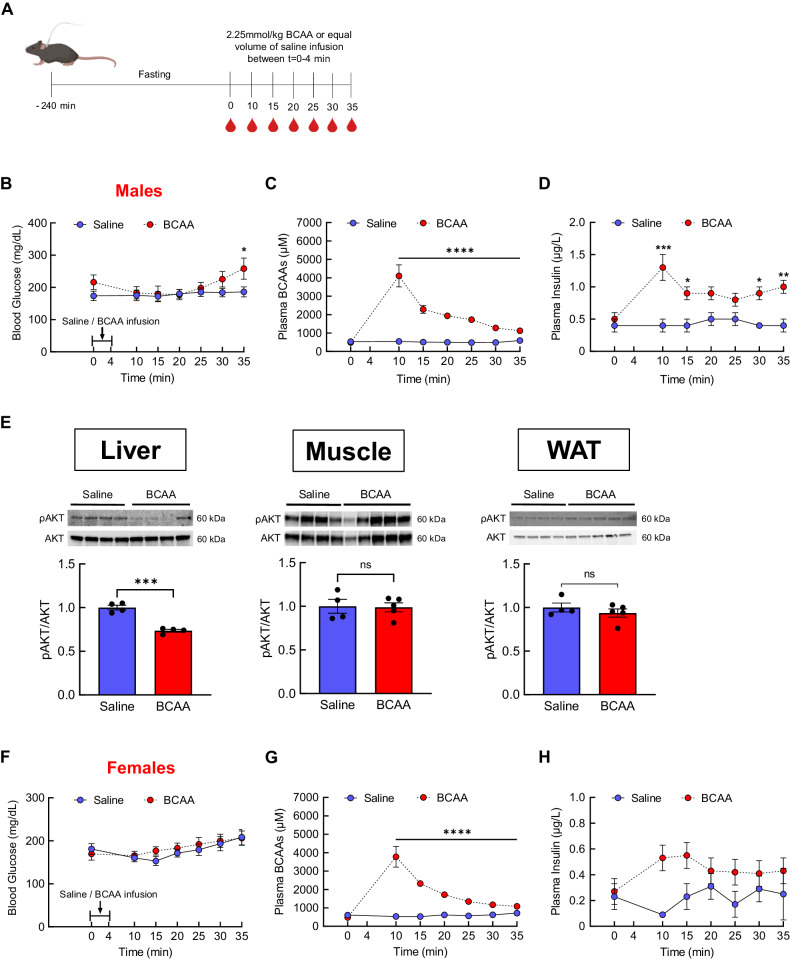
BCAA infusion acutely raises blood glucose. **A** Experimental design, **B** blood glucose, **C** plasma BCAAs, and **D** plasma insulin following either saline (*n* = 8) or BCAA infusion (*n* = 10) through jugular catheter during 30 min period in regular chow-fed male mice. **E** Western blot data. A separate cohort of male mice that were pre-treated with acute saline or BCAAs were injected with insulin (0.75 IU/kg i.p.; *n* = 4 saline; *n* = 5 BCAA) and euthanized 1 h later to measure protein expression of phosphorylated AKT (pAKT) as a marker of insulin signaling in liver, muscle, and epididymal white adipose tissue. **F** Blood glucose, **G** plasma BCAAs, and **H** plasma insulin after either saline (*n* = 8) or BCAA infusion (150 mM; *n* = 8) in regular chow-fed females. Mean ± SEM; *P < 0.05; **P < 0.01; ***P < 0.001; ****P < 0.0001.

BCAAs are shown to affect pancreatic hormones such as insulin and glucagon [45, 46]. Since blood glucose is very sensitive to these hormones, the dynamics of glycemic change are best captured through a repeated sampling method. To this end, a jugular catheter was implanted in mice to perform frequent blood glucose sampling (Experimental design; Fig. 1A). A single bolus of either saline or BCAAs was infused intravenously via the indwelling catheter in freely moving chow-fed mice, and blood glucose was monitored and blood was collected every 5 min through the catheter. The physiological dose of BCAAs employed in this study is equivalent to the amount of BCAAs present in a single meal of a mouse (details in Methods). Unlike saline-treated male mice that displayed a stable blood glucose throughout the experiment, the BCAA-treated group showed an increased blood glucose at *t* = 30 min and nearly a 20% rise compared to the saline group at *t* = 35 min (Time, *F*[2,31] = 9.1, *P* = 0.0007; Interaction, *F*[6,91] = 5.5, *P* < 0.0001; Fig. 1B). Plasma BCAAs sharply elevated at *t* = 10 min before steadily declining thereafter, but remained higher compared to those from the control male mice (Time, *F*[1,14] = 26.6, *P* < 0.0001; Treatment, *F*[1,11] = 159.6, *P* < 0.0001; Interaction, *F*[6,62] = 26.9, *P* < 0.0001; Fig. 1C). Interestingly, a simple BCAA infusion significantly raised plasma insulin by 200–260% that was maintained until the end of experiment (Interaction, *F*[1,108] = 54.7, *P* < 0.0001; Fig. 1D). The ability of BCAAs to acutely increase blood glucose in the face of higher plasma insulin points to a possible insulin resistance. Measurement of insulin-stimulated insulin signaling showed ~30% reduced protein expression of phosphorylated AKT (pAKT) in the liver, but not in white adipose tissue or muscle of BCAA-treated mice compared to that in saline-treated controls, indicating specifically hepatic insulin resistance (Fig. 1E). On the contrary, BCAA infusion in female mice did not result in any changes in blood glucose (Fig. 1F) or plasma insulin even though plasma BCAAs were indeed markedly elevated compared to those in the control group (Fig. 1G, H). To determine which one of the three BCAAs may have an acute, direct hyperglycemic effect, male mice were treated with a single bolus of either saline, leucine, isoleucine, or valine, and blood glucose was monitored for 1 h. While the treatment with any three individual amino acids was able to increase total plasma BCAAs (Supplementary Fig. 1A), only valine was found to acutely and significantly elevate blood glucose by nearly 100% (*F*[3,14] = 4.3, *P* < 0.02; Supplementary Fig. 1B, C). Altogether, these findings suggest that BCAAs can acutely raise blood glucose that is possibly mediated through hepatic insulin resistance, and that this is sex-specific.

### A single BCAA injection does not affect glucose homeostasis, but impairs the counterregulatory response to hypoglycemia

**Fig. 2:**
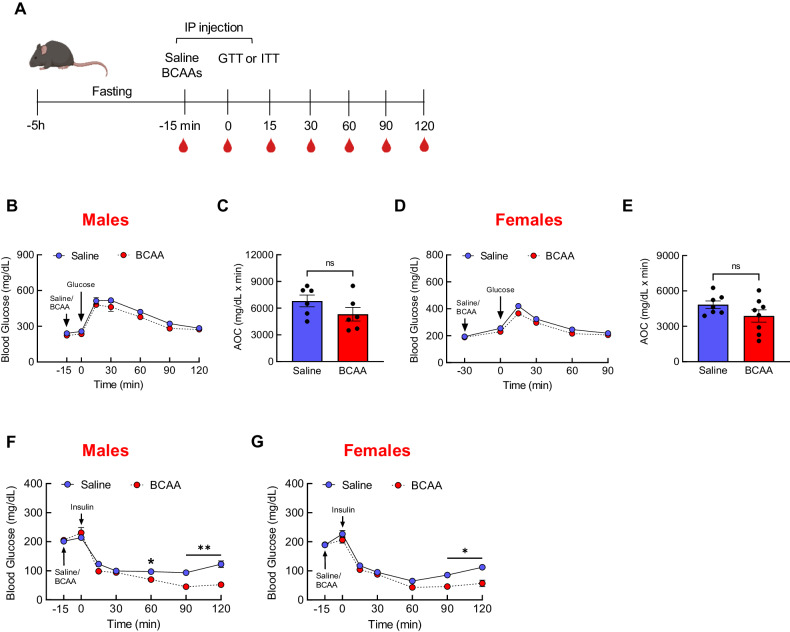
Pre-treatment with BCAAs does not affect glucose tolerance or insulin sensitivity. **A** Experimental design. **B** Blood glucose during GTT (1.5 g/kg i.p.). **C** Glucose AOC after i.p. injection of either saline (*n* = 6) or BCAAs (225 mM; *n* = 6) in regular chow-fed male mice. **D** Blood glucose during GTT (1.5 g/kg i.p.). **E** Glucose AOC after i.p. injection of either saline (*n* = 7) or BCAAs (*n* = 8) in regular chow-fed female mice. **F** Blood glucose during ITT (0.75 IU/kg i.p.) following i.p. injection of either saline (*n* = 5) or BCAAs (225 mM; *n* = 5) in male mice or **G** female mice (*n* = 6/group). Mean ± SEM; *P < 0.05; **P < 0.01.

The hyperglycemic effects of BCAAs suggested that they may be able to impair glucose homeostasis, thus we conducted an intraperitoneal glucose tolerance test (ipGTT) in mice injected with either saline or BCAAs (Experimental design; Fig. 2A). Compared with saline injection, pre-treatment with BCAAs surprisingly did not affect glucose excursion in both male (Fig. 2B, C) and female mice (Fig. 2D, E). Insulin tolerance test (ITT) also revealed no differences in the slope of blood glucose lowering and hence insulin sensitivity following saline or BCAA injection in both males and females (Fig. 2F, G). However, BCAA-treated mice failed to rebound blood glucose beyond *t* = 60 min unlike the control mice, indicating possible impairment in counterregulatory response. Detailed analyses in males during ITT demonstrated a significant increase in plasma BCAAs in BCAA-injected group (Time, *F*[2.6,20.5] = 15.9, *P* < 0.0001; Treatment, *F*[1,8] = 2.79, *P* = 0.13; Interaction, *F*[4,32] = 9.63, *P* < 0.0001; Supplementary Fig. 2A). Plasma insulin increased in both saline and BCAA groups as expected (Time, *F*[1.67,11,3] = 38.32, *P* < 0.0001; Interaction, *F*[4,27] = 2.22, *P* = 0.093; Supplementary Fig. 2B), and there was no sign of BCAA-induced change in endogenous insulin release as evidenced by similar plasma C-peptide levels during ITT (Supplementary Fig. 2C). Interestingly, while saline group temporarily increased plasma levels of the counterregulatory hormone glucagon at *t* = 15 min in response to the large drop in blood glucose, BCAA group similarly elevated plasma glucagon but maintained high levels throughout ITT (Time, *F*[4,28] = 14.4, *P* < 0.0001; Treatment, *F*[1,7] = 2.19, *P* = 0.18; Interaction, *F*[4,28] = 3.89, *P* = 0.012; Supplementary Fig. 2D). This was in agreement with higher excursion of plasma corticosterone, another counterregulatory hormone critical for restoring euglycemia (*t* = 2.79, *P* < 0.05; Supplementary Fig. 2E, F). These findings suggest that BCAAs do not acutely alter glucose homeostasis in chow-fed mice, but rather are capable of disrupting counterregulatory response to hypoglycemia, possibly by decreasing sensitivity to glucagon and corticosterone.

### BCAAs acutely impair whole-body insulin sensitivity

**Fig. 3:**
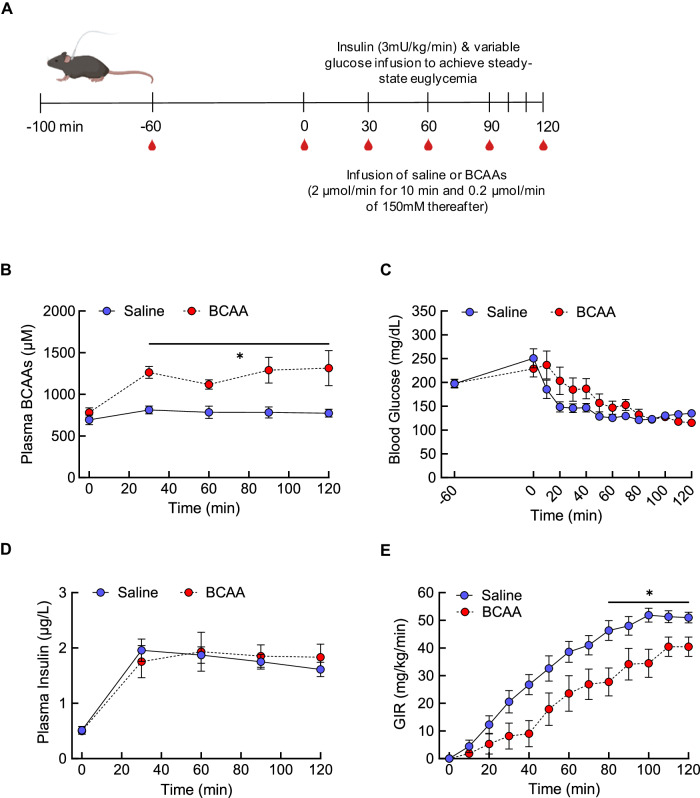
BCAAs acutely impair whole-body insulin sensitivity during pancreatic clamps. **A** Experimental design of hyperinsulinemic–euglycemic clamps. **B** High plasma BCAAs during constant BCAA infusion. **C** Blood glucose achieving euglycemia during steady state. **D** Confirmation of hyperinsulinemia during clamps. **E** Glucose infusion rate (GIR) during clamps and constant infusion of either saline (*n* = 7) or BCAAs (*n* = 7) in 4 h-fasted male mice. Mean ± SEM; *P < 0.05.

Our observations that plasma insulin rises sharply and comes down gradually during ITT, which leads to BCAA fluctuations, unfortunately do not offer a clear picture on the role of BCAAs in insulin sensitivity in an acute setting. To test this in a more controlled manner, we performed hyperinsulinemic–euglycemic clamps in mice that were implanted with a jugular catheter (Experimental design; Fig. 3A). Pancreatic clamp is the “gold-standard” method to assess whole-body insulin sensitivity which is accomplished by inducing constantly high circulating insulin and infusing glucose at variable rates to maintain euglycemia. Therefore, the more insulin-sensitive an animal is, the higher glucose infusion rate (GIR) that is required to maintain euglycemia; vice versa, the more insulin-resistant an animal is, the lower GIR that is necessary to keep euglycemia. With primed BCAA infusion at 2 µmol/min for 10 min followed by 0.2 µmol/min thereafter for 110 min, we were able to sustain high plasma BCAA levels throughout the clamp period (Time, *F*[1.84,22.1] = 6.53, *P* < 0.01; Treatment, *F*[1,12] = 13.42, *P* < 0.01; Interaction, *F*[4,48] = 3.17, *P* < 0.05; Fig. 3B). Our results demonstrate that in order to ensure stable blood glucose at euglycemia (Fig. 3C) in response to hyperinsulinemia during the clamps (Time, *F*[4,62] = 16.82, *P* < 0.0001; Treatment, *F*[1,62] = 0.065, *P* < 0.78; Interaction, *F*[4,62] = 0.303, *P* = 0.87; Fig. 3D), BCAA-treated male mice had significantly lower GIR especially during the steady state compared to saline-treated controls (Time, *F*[3.06,36.7] = 77.41, *P* < 0.0001; Treatment, *F*[1,12] = 7.67, *P* < 0.05; Interaction, *F*[12,144] = 2.36, *P* < 0.01; Fig. 3E). This was not observed in female mice (Supplementary Fig. 3A, B) which is in line with the lack of BCAA-induced glucose dysregulation in females. These findings suggest that BCAAs can acutely disrupt whole-body insulin sensitivity that may at least partly explain their ability to impair glycemic control.

### A BCAA-lowering compound BT2 effectively prevents the rise of plasma BCAAs in male mice

**Fig. 4:**
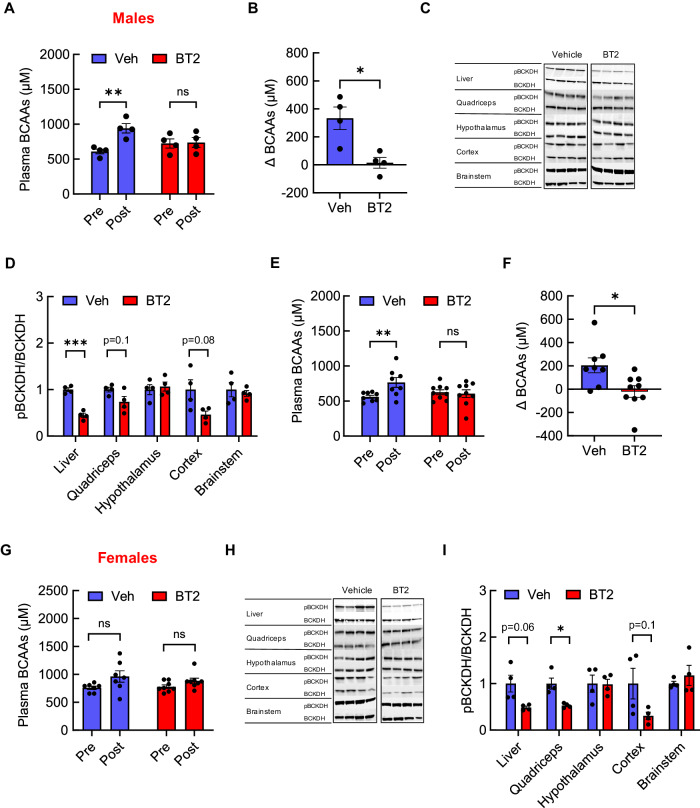
BT2, a BCAA-lowering compound, acutely prevents rise in circulating BCAAs in male mice. Vehicle or BT2 (40 mg/kg) was administered in mice fasted for 5 h. **A** Plasma BCAAs before and after treatment. **B** Changes in plasma BCAAs. **C** Western blots for BCKDH protein and its phosphorylation state in the liver, quadriceps muscle, hypothalamus, cortex, and brainstem. **D** Western blot analysis in male mice following i.p. injection of either vehicle (*n* = 4) or BT2 (*n* = 4). **E** Plasma BCAAs. **F** Changes in plasma BCAAs in male mice after intravenous infusion of either saline (*n* = 8) or BT2 (*n* = 9) in 3 h-fasted male mice. **G** Plasma BCAAs (*n* = 8/group), **H** western blots for BCKDH protein in tissues, and **I** western blot analysis in female mice after i.p. injection of either vehicle (*n* = 4) or BT2 (*n* = 4). Mean ± SEM; *P < 0.05; **P < 0.01; ***P < 0.001.

Glucose dysregulation and insulin resistance following a single exposure to BCAAs tempted us to test if an acute lowering of plasma BCAAs would exert positive metabolic effects. Indeed, chronic restriction of BCAAs or isoleucine alone as a means to reduce systemic BCAAs has been shown to improve glucose homeostasis and insulin sensitivity in animal models [28, 30, 37], but its acute effects are less understood. BT2, a small-molecule inhibitor of branched-chain α-keto acid dehydrogenase (BCKDH) kinase, was utilized to enhance BCAA catabolism [47]. As a result, BT2 effectively lowers plasma BCAAs when given for several days or weeks [32, 47, 48]. To first determine if a single bolus of BT2 can reduce plasma BCAAs, 5h-fasted male mice were injected with either vehicle or BT2 (40 mg/kg i.p.) and BCAAs were measured 2 h after in the absence of food. As opposed to markedly elevated plasma BCAAs compared to the baseline in vehicle-treated mice due to prolonged fasting (Interaction, *F*[1,15] = 7.8, *P* = 0.01; Fig. 4A), a single BT2 injection was sufficient to completely prevent the rise of BCAAs such that the change between pre- vs. post-treatment was significantly less in BT2 group compared to that in the vehicle group (Fig. 4B). As expected, this outcome is likely due to an efficient BCAA breakdown most notably in the liver, as indicated by decreased BCKDH inactivity index (i.e., ratio of phosphorylated BCKDH to total BCKDH; Fig. 4C, D). Higher BCAA catabolism was also observed in quadriceps muscle and brain cortex, although it was not statistically significant. Since BT2 administration route was different from that for BCAAs, we sought to test the effects of an acute intravenous BT2. Our results confirmed that intravenous BT2 infusion, similar to intraperitoneal injection, also blocked any increase in plasma BCAAs that was apparent in the control group (Time, *F*[1,15] = 5.2, *P* < 0.05; Interaction, *F*[1,15] = 7.8, *P* < 0.05; Fig. 4E, F). On the other hand, the same BT2 effects were not observed in female mice (Fig. 4G) which was interestingly independent of induced BCAA catabolism in the liver, muscle, and brain cortex (Fig. 4H, I). Collectively, these findings suggest that BT2 can acutely resist prolonged fasting-induced BCAA increase by primarily stimulating hepatic BCAA breakdown, and this effect is restricted to male mice.

### BT2 acutely restores glucose homeostasis in male mice on a HF diet

**Fig. 5:**
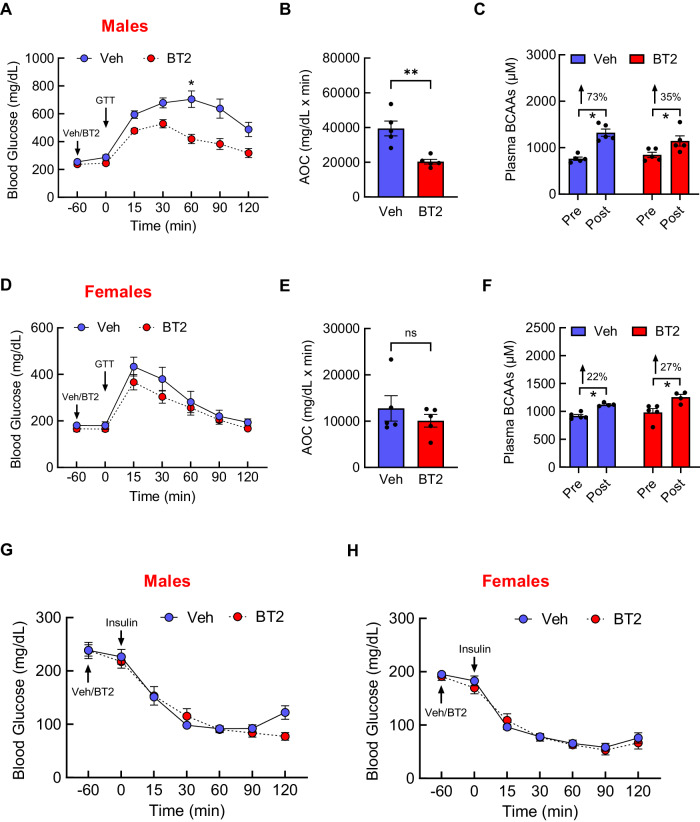
A single injection of BT2 is sufficient to improve glucose homeostasis in diet-induced obese mice. Mice were fed a high-fat diet for 8 weeks. **A** Blood glucose during GTT following pre-treatment with either vehicle (*n* = 5) or BT2 (40 mg/kg i.p.; *n* = 5) in 5 h-fasted male mice. **B** Blood glucose AOC. **C** Plasma BCAAs before and after treatments. **D** Blood glucose during GTT following pre-treatment with either vehicle (*n* = 5) or BT2 (40 mg/kg i.p.; *n* = 5) in 5 h-fasted, diet-induced obese female mice. **E** Blood glucose AOC. **F** Plasma BCAAs before and after treatments. **G** Blood glucose during ITT after pre-treatment with either vehicle (*n* = 5) or BT2 (*n* = 5) in diet-induced obese male mice or **H** female mice. Mean ± SEM; *P < 0.05; **P < 0.01; ***P < 0.001; ****P < 0.0001.

Next, obesity-related glucose impairment is associated with high plasma BCAAs [21, 23, 25, 26], thus we tested if BT2 in diet-induced obese mice can at least partly normalize glucose homeostasis through affecting BCAAs. Mice were rendered obese on a 60% HF diet for 8 weeks (Supplementary Fig. 4) before they underwent ipGTT with either vehicle or BT2 pre-treatment. As shown in Fig. 5A, B, a single injection of BT2 was sufficient to significantly improve glucose tolerance in obese male mice (Time, *F*[2.26,18.11] = 67.8, *P* < 0.0001; Treatment, *F*[1,8] = 13.5, *P* < 0.01; Interaction, *F*[6,48] = 8.17; *P* < 0.0001). While BT2 did not lower plasma BCAAs, it was able to resist BCAA rise compared to vehicle treatment during GTT by more than twofold ( ↑ 73% Vehicle vs. ↑ 35% BT2; Fig. 5C). Glucose-improving effects were absent in regular chow-fed male mice (Supplementary Fig. 5A–C), suggesting that acute metabolic benefits of BT2 are specific to mice with the nutrient surplus. In contrary, there was no difference in glucose tolerance (Fig. 5D, E) or plasma BCAA levels (Fig. 5F) following vehicle or BT2 injection in HF-fed obese female mice. We also did not observe any differences in insulin sensitivity through ITT in both diet-induced obese male and female mice (Fig. 5G, H). These findings suggest that obesity-associated impairment in glucose homeostasis can be acutely and effectively reversed by BT2, specifically in male mice, without affecting insulin sensitivity.

### The ability of AgRP neurons to impair glucose tolerance requires BCAAs

**Fig. 6:**
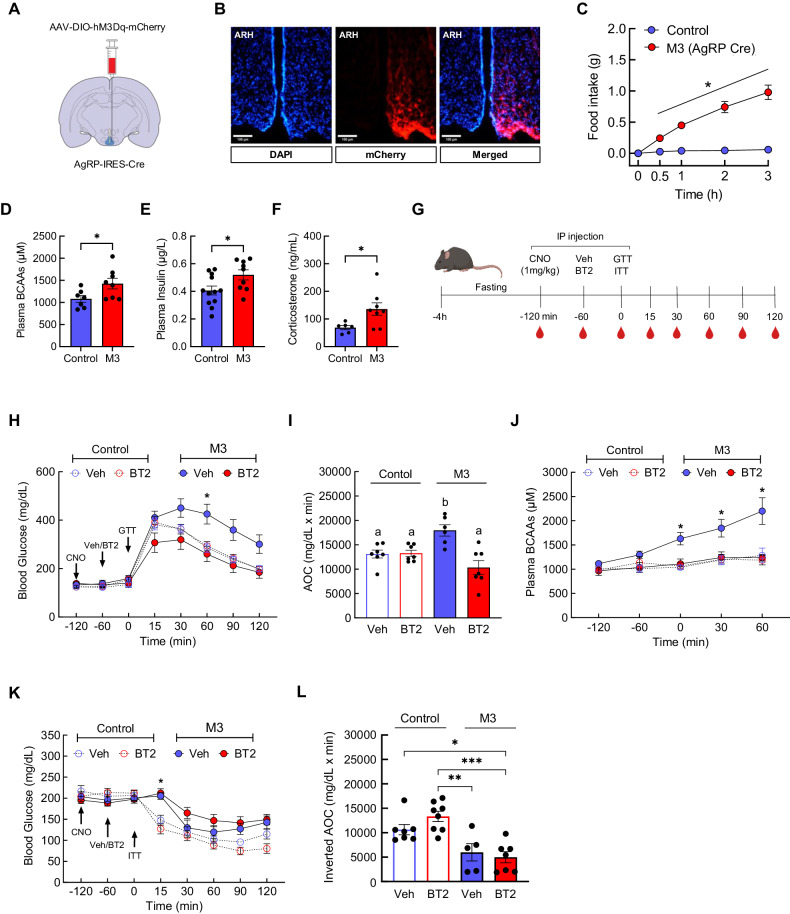
Acute stimulation of AgRP neurons disrupts glucose homeostasis primarily due to BCAAs. **A** Schematic shows unilateral injection of AAV encoding Cre-dependent hM3Dq-mCherry (DREADD; 400 nl) into the arcuate nucleus (ARH) of C57Bl/6J or AgRP-IRES-Cre male mice. **B** Representative image showing hM3Dq-mCherry-expressing AgRP neurons in AgRP-hM3Dq mouse at ×20 magnification. **C** Food intake measurement as a functional readout of DREADD expression in Control (*n* = 14) vs. AgRP-hM3Dq mice (M3; *n* = 13). **D** Plasma BCAAs, **E** plasma insulin, and **F** plasma corticosterone between mice without AgRP neuronal stimulation and with AgRP neuronal stimulation (*n* = 6–11/group). **G** Experimental design showing CNO injection to stimulate AgRP neurons, followed by vehicle or BT2 injection (40 mg/kg i.p.) and ipGTT (1.5 g/kg) or ipITT (0.75 IU/kg). **H** Blood glucose, **I** blood glucose AOC, and **J** Plasma BCAAs during GTT between Control vs. M3 mice (*n* = 6–7/group). **K** Blood glucose during ITT after vehicle or BT2 pre-treatment between Control vs. M3 mice (*n* = 5–8/group). **L** Inverted AOC of blood glucose during ITT. Mean ± SEM; *P < 0.05; **P < 0.01; ***P < 0.001.

Apart from peripheral organs such as liver, muscle, and fat, the hypothalamus in the brain is widely regarded as an important glucoregulatory center that becomes dysfunctional in obesity [49]. We and others [41, 50] have recently shown that an acute, optogenetic or chemogenetic stimulation of agouti-related protein (AgRP)-expressing neurons in the arcuate nucleus of the hypothalamus (ARH), similar to the hyperactive state in obesity, markedly elevates plasma BCAAs and impairs glucose homeostasis in mice. To test if high BCAAs are causative for this phenomenon, the ARH of either C57BL/6J or AgRP-IRES-Cre male mice on a regular chow diet was injected unilaterally with AAV containing stimulatory hM3Dq DREADD (Fig. 6A, B). Functional DREADD expression was demonstrated in AgRP-IRES-Cre mice as evidenced by significantly increased food intake following injection of the designer drug, clozapine-N-oxide (CNO, 1 mg/kg i.p.) at fed state, compared to that in the control group (Fig. 6C). Female mice were not examined due to their lack of responses to acute change in BCAAs in previous experiments. We also confirmed what we and others found previously [41, 50, 51] on markedly higher plasma BCAAs, insulin, and corticosterone upon acute stimulation of AgRP neurons (Fig. 6D–F). Next, following CNO injection, mice were treated with either a single dose of vehicle or BT2 (40 mg/kg i.p.) prior to GTT (Experimental design; Fig. 6G). BT2 did not affect glucose excursion in control mice (Fig. 6H, I). As expected, M3 group with acute stimulation of AgRP neurons displayed significantly impaired glucose tolerance compared to the control group, but this was completely reversed in M3 mice pre-treated with BT2. In parallel, plasma BCAAs continuously increased during GTT in mice with AgRP neuronal activation, but they were completely normalized in BT2-injected mice (Fig. 6J). On the other hand, while M3 mice displayed insulin resistance during ITT compared to the control mice, pre-treatment with BT2 was not able to correct it (Fig. 6K, L). Altogether, these findings suggest that hyperactivation of AgRP neurons acutely impairs glucose homeostasis through BCAAs.

## Discussion

In modern days, a growing number of health-conscious individuals aim to stay away from a high-carbohydrate or high-fat foods, and instead choose to follow a diet rich in quality proteins that come from lean meat, poultry, fish, and nuts, all of which contain a high amount of BCAAs. Also considered as one of the most popular dietary supplements today, BCAAs are implicated in physical fitness and are known to alleviate muscle-wasting conditions [9, 14]. However, recent evidence suggests that BCAAs are linked with obesity, insulin resistance, and diabetes [24–27]. Restriction of BCAAs improves glycemic control and metabolic health [37, 52, 53] whereas BCAA supplementation can impair glucose metabolism and insulin sensitivity [18, 21, 23, 29, 54]. Whether these are causally linked to direct or secondary effects of BCAAs are not clear from these studies due to the nature of the long-term interventions employed. With the help of in vivo physiological techniques including catheter-guided frequent sampling and hyperinsulinemic–euglycemic clamps, our current findings demonstrate that acute manipulation of systemic BCAAs is sufficient to disrupt glucose homeostasis and insulin sensitivity in mice, thus providing possible mechanistic insights into the detrimental outcomes following chronic BCAA supplementation observed in the literature. We further elucidated that acute central regulation of glucose homeostasis is primarily mediated by BCAAs.

A single infusion of BCAAs led to a small drop followed by a substantial increase in blood glucose in young C57Bl/6J male mice ( ↑ 20% from their baseline; ↑ ~50% from control group). The rise in plasma BCAAs starting from *t* = 10 min coincides with increased plasma insulin that was maintained until the end of the study. BCAAs, especially leucine, are known to act as an insulin secretagogue [55], and that explains why BCAA-infused group initially dropped blood glucose that is most likely driven by higher insulin. What captured our interest was the significant increase in blood glucose afterward despite sustained, higher plasma insulin compared to that in saline-infused mice. Two possible explanations exist for this phenomenon. First, since BCAAs can serve as an energy source, excess availability of BCAAs as a fuel may decrease glucose utilization which may in turn cause a rise in blood glucose. Indeed, a bolus infusion of a purely glucogenic amino acid valine was able to raise blood glucose compared to saline infusion. However, when we conducted GTT to assess glucose clearance, both male and female mice did not show any sign of glucose intolerance after receiving BCAAs. These results suggest that BCAAs most likely do not affect glucose utilization in an acute setting when supplemented exogenously. Second, since insulin is known to facilitate glucose clearance by promoting glucose uptake into tissues like muscle and white adipose tissue, BCAA-induced elevation in blood glucose in spite of hyperinsulinemia points to the possibility of insulin resistance. While we did not observe any changes in insulin sensitivity during ITT after BCAA injection, an accurate interpretation becomes difficult since circulating BCAAs do not remain elevated shortly after a bolus of insulin, most likely because of insulin’s ability to promote amino acid disposal. Hence, in order to keep constantly elevated BCAAs during the test period, BCAAs were infused through an indwelling jugular catheter in mice during hyperinsulinemic–euglycemic clamps, the *“gold-standard*” method for evaluating whole-body insulin sensitivity. By utilizing this physiological integrative technique, we were also able to maintain high plasma insulin that was not achievable during ITT. Mice with BCAA infusion lowered GIR that is necessary to maintain euglycemia in the face of hyperinsulinemia, indicating decreased insulin sensitivity. It is worth noting that short-term effects of amino acids on insulin sensitivity have been investigated through pancreatic clamps in several human studies [56–61], but the results are inconsistent. Infusion of amino acid mixture including BCAAs during hyperinsulinemic–euglycemic clamps decreased GIR which is indicative of insulin resistance [57–59, 62], most likely via impairing peripheral glucose disposal without affecting endogenous glucose production [59]. However, a recent study by Burgos and colleagues did not observe any effects of hyperaminoacidemia on insulin sensitivity in men [61]. These conflicting reports could be due to the differences in the composition of amino acid mixtures, circulating amino acid levels achieved during the clamps, and/or the magnitude of hyperinsulinemia used in these studies. A small cohort study by Everman et al. examined the effects of a short-term increase in BCAAs exclusively during hyperinsulinemic–euglycemic clamps and did not reveal any appreciable changes in insulin sensitivity [60]. A clear interpretation from this elegant study is somewhat limited by the low sample size and the inability of the control group to reach a statistically significant hyperinsulinemia. One potential reason for not observing insulin resistance that was demonstrated by earlier studies, as the authors noted, could be that the saline control group was infused with BCAAs to maintain basal plasma BCAA levels during hyperinsulinemic clamps whereas other studies allowed hyperinsulinemia to drop plasma BCAAs below the baseline. This may have created a greater glucose disposal in the control group compared to the group infused with amino acid mixtures, leading to the conclusion that acute hyperaminoacidemia impairs insulin sensitivity. While that may be the case, our control mice were also able to maintain basal BCAAs during the clamps, yet displayed a clear increase in GIR compared to BCAA-infused mice. These results support the notion that acute BCAAs can elevate blood glucose by inducing insulin resistance. The precise nature of insulin resistance induced by BCAAs in our current study, i.e., the inability of insulin to suppress endogenous glucose production and/or enhance glucose disposal, remains unknown. Impaired insulin signaling after a single BCAA injection specifically in the liver, as evidenced by significantly reduced pAKT, suggests a possible defect in insulin’s ability to suppress hepatic glucose production that could explain higher blood glucose. Incorporating a tracer dilution technique by infusing a labeled glucose isotope during pancreatic clamps will provide us a clear picture. Chronic BCAA supplementation has been shown to induce insulin resistance in animal models primarily by lowering insulin-stimulated glucose uptake [21, 63]. The proposed mechanisms include hyperactivation of mTOR [21], reduction of AKT by its ubiquitination [63], and excess accumulation of valine metabolite, 3-hydroxy-isobutyrate (3-HIB), that allows for efficient fatty acid uptake to impair insulin action in muscle [18]. Whether or not similar mechanisms are responsible for insulin resistance in an acute setting is yet to be explored.

It is interesting that unlike saline-treated group, both BCAA-injected male and female mice were not able to recover from hypoglycemia during ITT. Our findings are in agreement with an earlier study in which leucine administration substantially prolonged the insulin-induced hypoglycemia in dogs as evidenced by lower arteriovenous glucose difference during the glucose recovery phase [64]. Although the hypoglycemic effect of leucine has been suggested to be due to its stimulating effect on insulin secretion [55], we did not observe any changes in plasma insulin or C-peptide excursions between control vs. BCAA groups. This suggests that the hypoglycemic effect of BCAAs observed in our current study was most likely not due to their action as insulin secretagogue but rather due to their inhibitory action on counterregulatory responses. Since hypoglycemia persisted in spite of higher glucagon and corticosterone, perhaps BCAAs can impair tissue sensitivity to these glucose-restoring responses. The underlying mechanisms as well as the role of other counterregulatory hormones such as epinephrine and growth hormone in this phenomenon require further investigation.

Lowering plasma BCAAs via chronic dietary BCAA restriction has been shown to improve insulin sensitivity although the exact mechanisms remain unclear [28, 30, 37]. Here we used a pharmacological strategy to lower BCAAs and revealed that a single injection of BT2, a potent activator of BCAA catabolism, can prevent fasting-induced rise in plasma BCAAs as observed in our earlier study [41]. While having no effects in lean mice, pre-treatment with BT2 significantly improved glucose homeostasis in diet-induced obese mice. In support of our results, recently two independent groups [33, 65] also demonstrated improved glucose tolerance in diet-induced obese mice following acute BT2 treatment at a higher dose (100 mg/kg). Using ITT, we did not detect any differences in insulin sensitivity after BT2 injection in diet-induced obese mice, whereas Bollinger and colleagues reported enhanced insulin sensitivity as measured by hyperinsulinemic–euglycemic clamps [33]. This could be due to differences in the physiological techniques or much higher dose of BT2 used in their study (40 mg/kg vs. 100 mg/kg). Similar to clinical studies, a number of rodent studies including our own demonstrated markedly higher plasma BCAAs in mice with HF-fed diet-induced obesity [23, 36, 66–68]. Since we and others have previously shown that over-stimulation of hypothalamic AgRP neurons, as evident in obesity [69], disrupts not only glucose homeostasis but also elevates circulating BCAA levels [41, 50], it was tempting to determine if the impaired glucose metabolism is largely mediated by high BCAAs. Here we were able to chemogenetically activate AgRP neurons and reproduce the results of higher plasma BCAAs. While the underlying mechanism is not clear, the Bruning group has recently demonstrated induction of hepatic autophagy and enhanced glucocorticoid action in mice with optogenetic stimulation of AgRP neurons [50]. Both glucocorticoids and autophagy are known to promote protein degradation [70–72] that could very well contribute to elevate plasma BCAAs. More importantly, mice with acute AgRP neuronal stimulation displayed markedly impaired glucose tolerance, but blocking the rise of BCAAs by pre-treating with BT2 completely normalized it. These results suggest that the brain control of glucose metabolism, at least in the acute setting, is dependent on BCAAs. Performing oral GTT and/or meal tolerance test would help determine the role of acute BCAAs in glucose homeostasis in a more physiological setting. In addition, testing if acutely suppressing AgRP neuronal activity in diet-induced obese mice improves glucose metabolism through BCAAs would further support the novel CNS-driven glucoregulatory pathway.

Sex differences in the effect of BCAAs on glucose homeostasis were clearly demonstrated in our study. Unlike male mice, female mice neither showed any rise in blood glucose after BCAA infusion, any changes in whole-body insulin sensitivity, nor significantly improved glucose metabolism after BT2 injection. This may be relevant to the differences in BCAA catabolic regulation between males and females, as shown earlier, mostly due to estrogen and the diurnal variation in regulating BCKDH enzyme [73]. Consistent with this, our previous study also found lower hepatic BCKDH in obese and diabetic men compared to healthy men, but not in women [74]. Future studies should focus on understanding the mechanism of sex differences in BCAA homeostasis and their impact on glycemic control and insulin sensitivity.

In conclusion, our study establishes the direct acute effect of BCAAs on glycemic control and insulin sensitivity that is largely independent of changes induced by chronically elevated BCAAs. These findings thus provide support for repurposing BCAA-lowering compounds to treat obesity and diabetes-related metabolic dysfunctions with consideration of potential sex differences in BCAA effects. Moreover, our findings that impaired glucose metabolism by AgRP neurons is mediated by BCAAs shed light on a novel mechanism underlying the brain control of glucose homeostasis.

### Supplementary information

Supp Figs 1–5

## Data Availability

The data that support the findings of this study are available from the corresponding author upon reasonable request.
