# Supplementary material for: BCAAs acutely drive glucose dysregulation and insulin resistance: role of AgRP neurons
Source: Nutr Diabetes. 2024 Jun 6;14:40. doi: 10.1038/s41387-024-00298-y (PMC11156648; doi:10.1038/s41387-024-00298-y)
Supplement: Supplementary file 1 — Supp Figs 1–5 [file 41387_2024_298_MOESM1_ESM.pdf]

Supplementary Information

These materials contain additional data for individual BCAA-injection study (Supp Fig. 1), counterregulatory responses during ITT (Supp Fig. 2), hyperinsulinemic clamp data for female mice (Supp Fig. 3), Body weight and composition in HF-fed mice (Supp Fig. 4), and BT2 effects during GTT on chow-fed male mice (Supp Fig. 5).

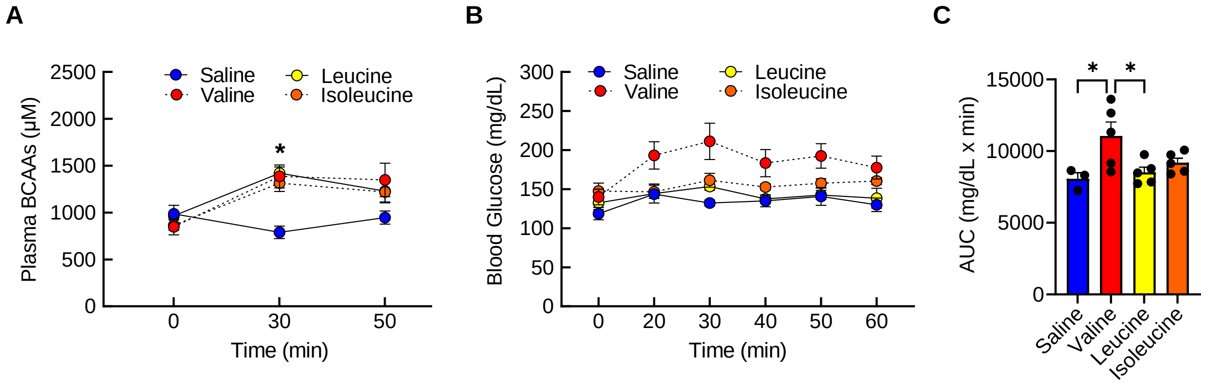

Supp Fig. 1. Valine acutely elevates blood glucose. **A)** Plasma BCAAs, **B)** Blood glucose, and **C)** AUC of blood glucose excursion following a single ip injection of either saline (n=3), leucine (n=5), isoleucine (n=5), or valine (n=5) at a concentration of 112.5mM, 56.25mM, and 56.25mM, respectively, in 2.5h-fasted mice. Mean±SEM; * p<0.05

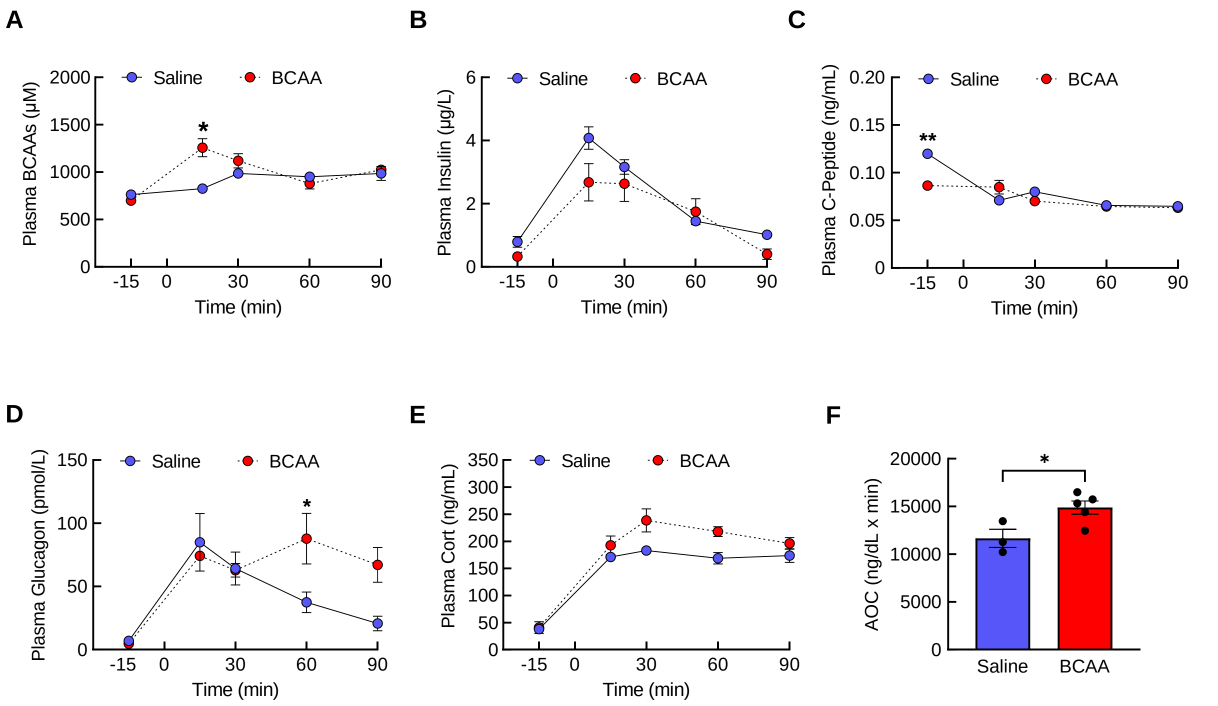

Supp Fig. 2. A single BCAA injection increases release of counterregulatory hormones during ITT. **A)** Plasma BCAAs, **B)** Plasma insulin, **C)** Plasma C-peptide, **D)** Plasma glucagon, **E)** Plasma corticosterone, and **F)** AOC for corticosterone in male mice during ITT after pre-treatment with either saline (n=5) or BCAAs (225mM; n=5). **E-F)** Due to the lack of plasma for corticosterone measurement, three mice were included for saline group. Mean±SEM; * $p<0.05$

A

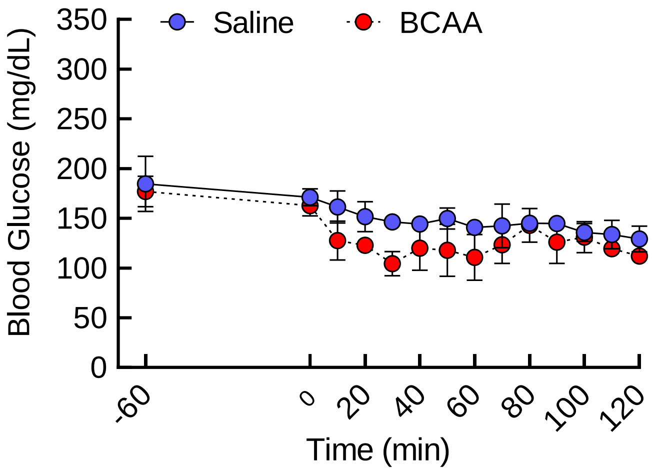

B

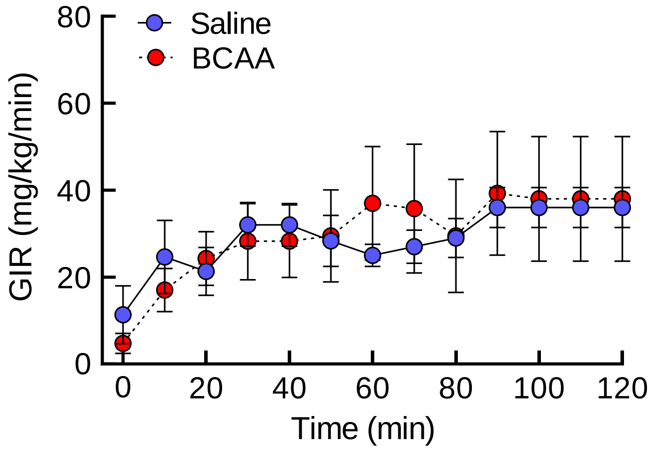

Supp Fig. 3. Acute BCAAs do not affect whole-body insulin sensitivity in female mice. **A)** Maintenance of euglycemia during steady state. **B)** Glucose infusion rate (GIR) during clamps and constant infusion of either saline (n=3) or BCAAs (n=4) in 4h-fasted female mice. Data are expressed in Mean±SEM.

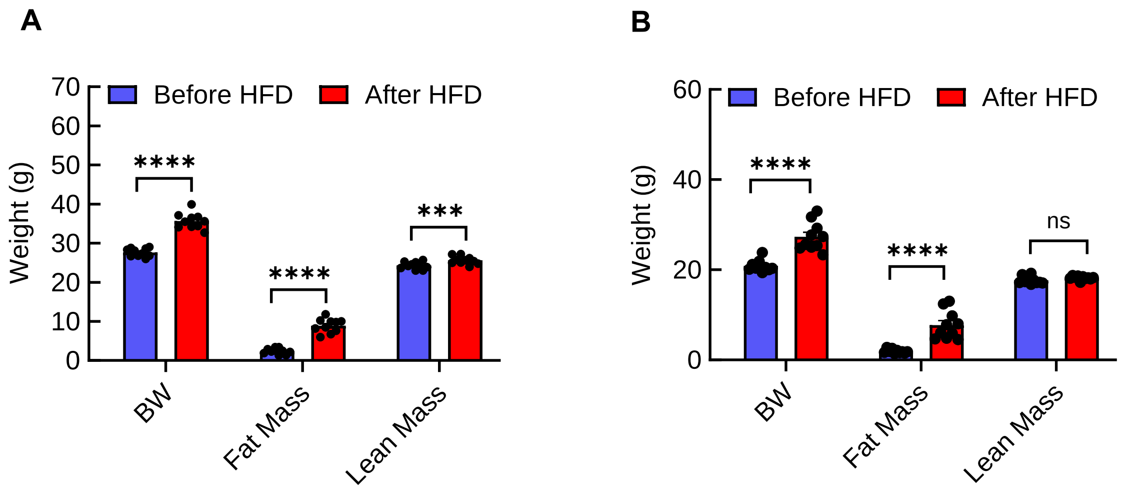

Supp Fig. 4. High-fat diet increases body weight and fat mass in mice. **A)** Measurement of body weight and body composition (fat and lean mass) in male mice before and after 8 weeks of HF feeding. **B)** Body weight and body composition (fat and lean mass) in female mice before and after 8 weeks of HF feeding. N=10/group; Mean±SEM; ***p<0.001, ****p<0.0001, ns=not significant

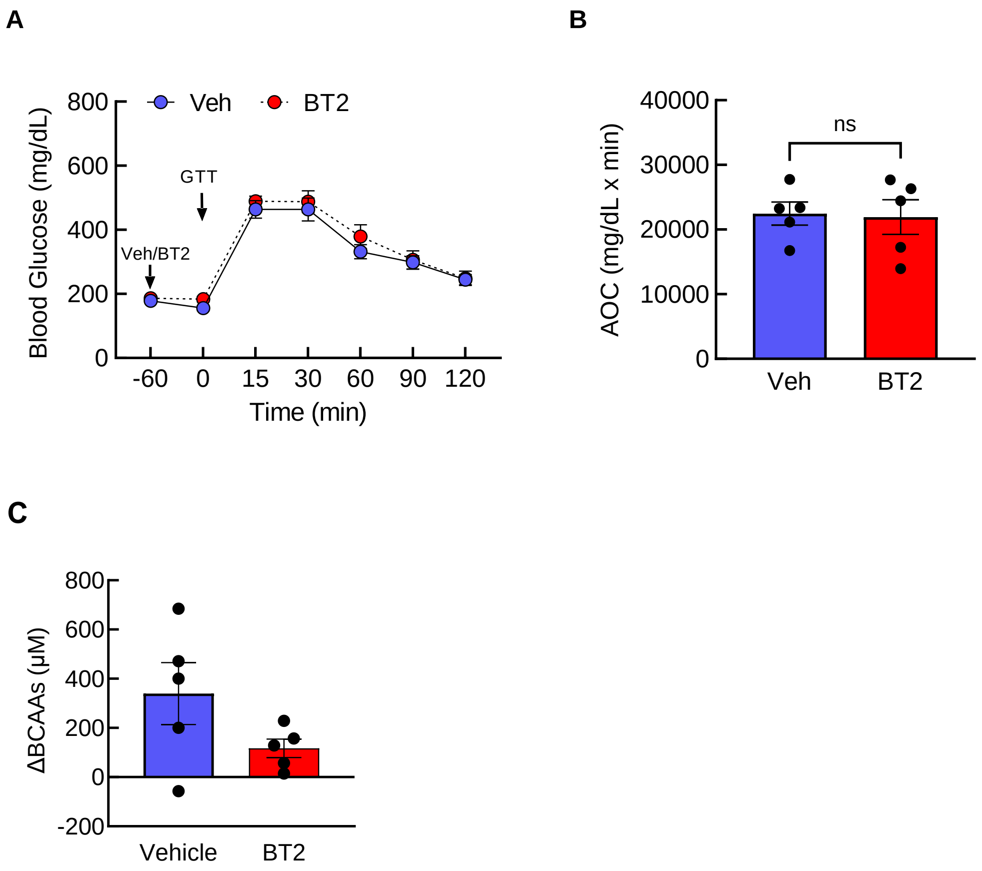

Supp Fig. 5. BT2 acutely does not alter glucose homeostasis in regular chow-fed lean mice. **A)** Blood glucose during GTT following pre-treatment with either vehicle (n=5) or BT2 (40mg/kg ip; n=5) in 5h-fasted male mice. **B)** Blood glucose AOC. **C)** Change in plasma BCAAs between pre- vs. post-treatment. Mean±SEM; ns=not significant
